# Human development IV: The Living Cell has Information-Directed Self-Organisation

**DOI:** 10.1100/tsw.2006.177

**Published:** 2006-09-07

**Authors:** Søren Ventegodt, Tyge Dahl Hermansen, Trine Flensborg-Madsen, Maj Lyck Nielsen, Birgitte Clausen, Joav Merrick

**Affiliations:** ^1^ Quality of Life Research Center, Teglgårdstræde 4-8, DK-1452 Copenhagen K, Denmark; ^2^ Research Clinic for Holistic Medicine, Copenhagen, Denmark; ^3^ Nordic School of Holistic Medicine, Copenhagen, Denmark; ^4^ Scandinavian Foundation for Holistic Medicine, Sandvika, Norway; ^5^ Interuniversity College, Graz, Austria; ^6^ Vejlby Lokalcenter, Vejlby, Denmark; ^7^ National Institute of Child Health and Human Development, Jerusalem, Israel; ^8^ Center for Disability and Human Development, Faculty of Health Sciences, Ben Gurion University, Beer-Sheva, Israel; ^9^ Office of the Medical Director, Division for Mental Retardation Ministry of Social Affairs, Jerusalem, Israel

**Keywords:** holistic biology, theoretical biology, clinical holistic medicine, morphogenesis, ontogenesis, developmental biology, Denmark

## Abstract

In this paper, restricted to describe the ontogenesis of the cell, we discuss the processing of DNA through RNA to proteins and argue that this process is not able to transfer the information necessary to organize the proteins in the cell, but only to transfer the information necessary to form the shape of the proteins. We shortly describe the structure of the information carrying field recruited by the cells that we think is responsible for building the organelles and other cellular structures. We use the cells superior control of its cytoskeleton as an example of how the cell is using an informational field giving the positional information guiding all the local chemical processes behind the cell movement. We describe the information-directed self-organization in cells and argue that this can explain the ontogenesis of the cell. We also suggest the existence of an undiscovered phenomenon behind the information transmitting cell interactions. We conclude that during evolution the cell has developed into an information-guided self-organizing structure. The mystery we want to solve is: what is the mechanical cause and nature of biological information?

## INTRODUCTION

Caused by lack of understanding of the cell itself, the contemporary biological theory of ontogenesis does not explain how the information for determination and differentiation of cells is recruited. Using the existing theory of DNA as donor of all cellular information, it has not been possible to explain the powers controlling the cell ontogenesis. We believe that these powers are transmitted through positional information. For instance, cell movement and axon and dendrite excrescence could be caused by a superior control of the cytoskeleton through positional information, thus making it possible for the cell to induce *information-directed self-organization*.

We believe that, in general, the shapes of organelles (as demonstrated in *Tetrahymena*)[1]) are not formed by self-organization of proteins alone, although the self-organizing property of proteins are well known. We found from analysis of the overall morphological dynamics of, say, the cytoskeleton, that more information is needed than what the molecules themselves are able to provide. We propose ontogenesis and cell determination to be explained by the cells using information-directed self-organization. For example, the organelles and other cellular structures are built because the cells can recruit and apply complex information though an information-carrying field. In existing biological theory, it is particularly the determination and differentiation of the cells that are not accounted for, probably due to the lack of understanding of the cell itself.

Generally speaking, ontogenesis is a phenomenon that is poorly understood and makes a puzzle for established science. Based on existing knowledge, we conclude that modern theories are not able to explain ontogenesis. We propose that a holistic biology using holistic organizing principles is needed to explain this astonishing enigma of biology. We propose a holistic biological paradigm based on distribution of information through roomy fractal structures, which seems to be in much better accordance with the real structure of biological organisms and ecological systems. In this series of papers, we have chosen to split the description of ontogenesis into two parts: "the ontogenesis of the cell" and "the supracellular ontogenesis". This paper is restricted to describe only the ontogenesis of the cell and review much of the existing data on this matter.

## WHAT WE THINK THE CELL IS

If the information of biological systems has to be understood, it is obvious that we must study how the complexity of the isolated biological system has been developed. This is the case for the development of all levels of the biological systems, such as the organization of the cell structure including organelles, Golgi apparatus, endoplasmatic reticulum (ER), etc.; as well as the supracellular structures (described in the next paper of this series). This process, creation of structure and organization, is called the morphogenesis; or a more comprehensive phrase, ontogenesis, the formation of the being. Fig. 1A and B show a generalized representation of the morphogenesis as complex bifurcation, where the different levels of organization are specified as M1, M2, etc. An example of such a structure in nature is a green Romanesco cauliflower (see an example of this on: http://www.fourmilab.ch/images/Romanesco/) We know very little about the mechanisms behind formation of shape, from molecular organization to cell organelles, and further to the superior levels. Therefore, we assume that the same principle stands behind the formation of shape on all biological levels. This counts for organization of organelles in cells, and cell organization to tissue, and this to the superior shape of organisms. The English philosopher, William of Occam (approx. 1285–1349) wrote, "when a phenomenon – fore instance, biological formation of shape – has to be explained, you have to use an absolutely minimum of assumptions" (Occam's razor[2,3]). Therefore, the cell is involved in organization of the ontogenesis of all levels. In this paper, however, we specifically describe the cellular ontogenesis and give a theory for the cell.

## THE ONTOGENESIS OF THE CELL

The mechanisms behind organization and morphogenesis are completely uncomprehended. The organization of cells takes place at different levels from molecules to organelles that again organize to form the full cell. A good question is: What kind of powers is the cell organizing on these subcellular levels? Based on the existing knowledge concerning DNA as storage of information, it is obvious to imagine that DNA-information is responsible for the organization of the cell. The transfer of information from DNA to proteins is carried out through a straight pathway, from the DNA through the RNA to proteins, where the

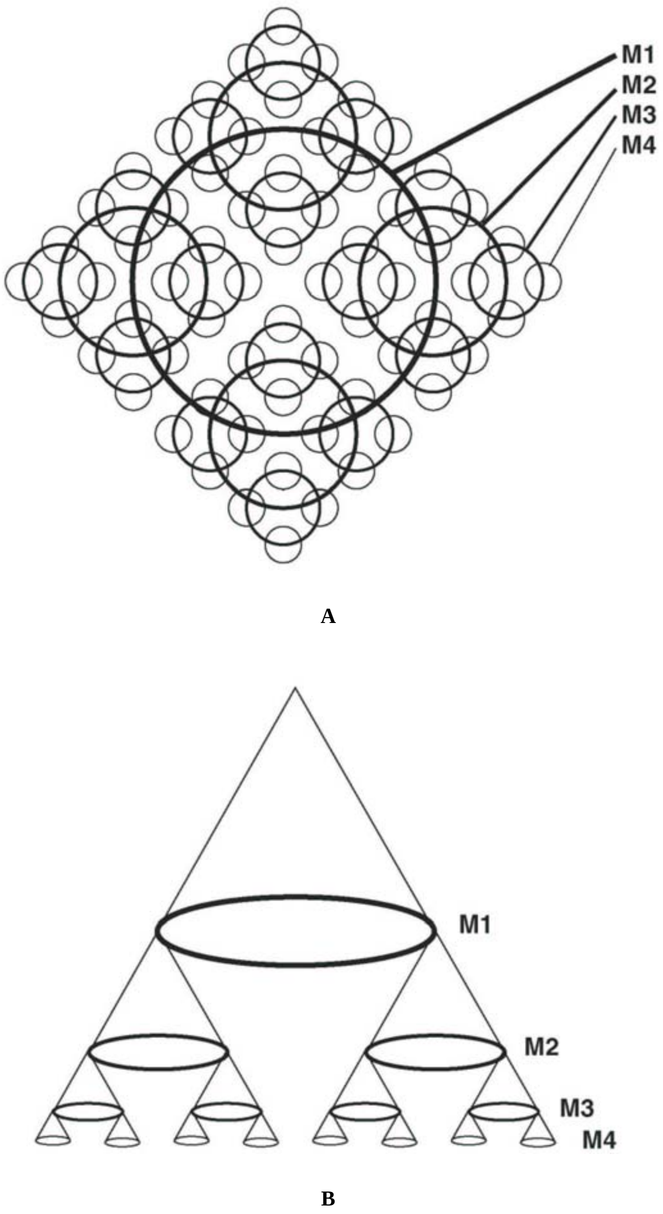

**FIGURE 1.** This figure gives a generalized representation of the morphogenesis as complex bifurcation. First, the zygote is created. This divides — eventually only by division of the core as with the insects — to form a heap of cells. This again divides (as, for instance, with *Hydra*) and when the new group of cells are suitable enough (variegating between different species), it performs its first organization corresponding to the superior construction of the organism (establishment of the germinal layer). After this, the embryo begins to grow and the germinal layers are divided into organ systems. These again, are divided into organs that again are divided into theirs structures on many levels. This goes on until all details of the organism are specified. All these levels of organization are specified in A and B as M1, M2, etc. (http://www.fourmilab.ch/images/Romanesco/)

RNA translates the DNA code to amino acids that again are gathered to form the proteins of the cell cytoplasm[1]. Thus, the DNA is merely able to transfer the information for the shape of the proteins, not to transfer the information necessary to organize the proteins in the cell. Consequently, it might be that the organizing mechanism comes from an unknown source. The mechanisms behind cell (as well supracellular) organization and morphogenesis thereby seem to be very difficult to describe in terms of a conventional (particular) molecular biological frame of reference. It is very interesting that while the details of biological mechanisms as the replication, transcription, and translation are very well described in molecular biological terms, the regulation of the eukaryotic gene expression — logically, a step in the morphogenesis — is completely uncomprehended, even though it has been thoroughly investigated through decades. Therefore, it seems that principles not yet known have to be discovered (this was also the position of Schrødinger[4]).

The affinity of proteins is directed against themselves and each other, giving a kind of simple self-organization as, for instance, virus capsules[1]. Here, it is tempting to suppose that simple self-organization can explain the whole cell and its behavior. However, organelles that are 100–1000 protein diameters in diameter do not show well-ordered, repetitive structures in the size of 10 protein diameters (common micrographs results). This is, for example, seen concerning the difference in mitochondria shape that depends on the type of cells[1], which do not support the simple self-organization idea. The viscous structure that would be caused by this kind of organization conflicts with the great mobility that is characteristic for the organelles. Below, we will discuss a number of concrete phenomena, not explainable by conventional knowledge, but able to enlighten the principles of cell-organization explained by our model.

## The Shape of Organelles is not Formed by Self-Organization of Proteins

The formation of virus capsule shape takes place through self-organization of proteins[1]. A similar process would be expected in our cells, but this is not the case. Even the polymerization of the simplest structures of the organelles, as actin and microtubules, seems to be the subject of superior and exact control from the cells[1]. For example, cells are able to send out microspikes, a kind of feelers, and draw them back again, if needed.

Generally speaking, cell organelles are shaped precisely after the needs of the specific type of cell. Examples of this are the endoplasmatic reticule, the Golgi apparatus, the vesicle, cell membrane systems, and mitochondrion, that take their shape after the specific type of cell[1]. This means that the shapes of organelles are often specified a lot more carefully than necessary in condition to the function it is ment to have. Seen in superior perspective, the mechanisms behind the design of organelle shape, mutual organelle movement, and precise mutual placement between the organelles (for example, vesicular transport to the right sack in the Golgi apparatus) are completely unknown.

At the Tetrahymenae, the cell structure is not decided by the proteins they consist of; this could be a general phenomenon in the world of the eukaryotes: “To a first approximation ... *Tetrahyminae pyriformis* cells and *T. vorax* microstomes maintain the same morphology with different proteins, whereas *T. vorax* microstomes and *T. vorax* macrostomes maintain different morphologies with the same proteins”[5]. Also, the genetically variegating *Tetrahyminae* often have the same shape. This means that proteins have no superior meaning for the formation of shape in this. Therefore, it is hardly the proteins that give the protozoans their manifold and magnificent forms[6].

## The Cells of a Body are Almost Alike Concerning Population of Proteins

Regardless of which two of a body’s approximately 300 functionally different cells are chosen, they will have about 98% of the most ordinary proteins in common in concentrations variegating less then a factor 5, and only 2% or less of the proteins will variegate more in concentrations, or, only be expressed in one of the cells[1]. This indicates that the molecular differences between two randomly chosen cells are not qualitative, but quantitative. So, one single specific cell may be characterized on the expression of a lot of genes. With this, we think it does not seem plausible that cellular formation of shape should be a function of the relations of the protein mix. Consequently, we have no molecular explanation of the cells morphology.

## The Foundation of Cell Movement and Axon and Dendrite Excrescence

We assume that a superior control of the cytoskeleton through positional information causes cell movement and axon and dendrite excrescence. In spite of great steps forward in the research of the cytoskeleton, the mechanisms behind movements, such as phagocytosis, mitoses, saltatory movements of mitochondrion and vesicles, and migration of cells, are still not very well known[1] and cell movement is one of the most challenging problems for the modern molecular biology today[1].

Cell movement, for example in connection with fibroblasts, is extremely complex because the formation and degradation of a great amount of different structures happens at the same time. It is coordinated across a huge area of the cell, during the formation of a pseudopodium (a kind of foot drawing a cell forward). Likewise, the growth conus moves itself ahead, corresponding to the pseudopodium, drawing the axon instead of the cell after itself[7].

The following phenomenon indicates a connection between cell migration and axon excrescence. When a cell *in vitro* proliferates, the two daughter cells can often be seen as a mirror image of each other through longer time[1] (see the 1983 edition, from the introduction figure b, page XXXVii). When the axon proliferates through growth *in vitro*, the same kind of symmetrical mirror image can be observed[7]. This indicates that cell movement and axon excrescence could be influenced by superior inside powers of organization able directly to steer the cytoskeleton organization of the cell. This could also be the case for the dendrites, since different nerve cell dendrites of vertebrates *in vitro* are observed to outgrow on a way that is typical for this type of cells[7]. Since we know nothing about the mechanisms behind the distribution of information to the cytoskeleton, we choose to define this phenomenon as being mediated through positional information on cell level.

## DISCUSSION

## Information-Directed Self-Organization Explains the Ontogenesis of the Cell

The same molecules generally build different cells and their different structures. The different ways of organization may be due to different information. Containing 20% of protein, the cytoplasm may be understood as a liquid viscous crystalline phase, and the cytoskeleton that takes care of all movement and transport of vesicles in the cell may be considered as a more solid part of this phase. Self-organization that is known from the most simple molecular organization (for instance, virus capsules) may be assumed to be a principle that also can be seen in the cell, but self-organization in a liquid crystal phase has to be a lot more complicated than in simple solid systems. Apparently, it is obvious that supply of information is necessary to avoid the superior level sinking into a phase of chaos; try to imagine the pattern behind cell movement. For example, is the well-organized walk of a fibroblast an inconceivable well-organized incident? The cytoskeleton, in maybe a thousand different places, has to react chemically in ways that are different from the close neighboring areas. All this happens in an extremely coordinated manner. To get a picture of the capability of the cell in this respect, just think about the patterns behind mitosis or behind the internal organization of the skeletal muscle cell.

The information necessary for this organization can be outlined as a pattern imprinted to the biological system, and as we, as mentioned above, do not know any physical laws that are able to generate such complex patterns in a reproducible way, we may assume that an undiscovered naturale phenomenon exists behind the transmission of information.

## Cell Involvement in Morphogenesis

Information-transmitting interactions give information-directed self-organization in biological systems. When the information-transmitting interactions have given the information for the organization to the cells that thereby can organize themselves through complex behavior — as, for instance, cell movement and advanced reorganization of the cytoskeleton to desmosomes — these cells can be organized at a higher level through new information. By this, the information-directed self-organization that was able to explain the cell ontogenesis, is also able to explain the organization of the higher levels without problems[8]. As was the case for the molecular conditions compared to the organelles, it is the case for the cells that, despite their organization that apparently is quite often disordered with a 5- to 10-cell-diameter level, the organization always comes through to a higher level typically containing about 100 cell diameters (compare the liver lobulus, for example[9,10], when needed. However, the organization is never tighter than needed.

The mechanistic nature of the information system seems to be an undiscovered dimension of physical energy, a patterned aspect of it, so to speak, that has not yet been discovered in physics as it only manifests itself clearly in biological systems. You could call it an information-carrying global quantum field if you like. No doubt it must be continuous with the molecule orbitals of the proteins of the cells to regulate the activity of the proteins as we see it in cell walk, as discussed. The idea of cytoplasm as a liquid crystal has been suggested before[11,12].

## CONCLUSIONS

1. The regulation of eukaryotic gene expression is not understood at all, which is why we think it is necessary to look for another explanation than the current one. We do not think that the processing of DNA through RNA to proteins is able to transfer the information necessary to organize the proteins in the cell, but only to transfer the information necessary to form the shape of the proteins, which is why we think the organizing mechanism comes from a former unknown source, but is now able to be explained by our holistic way of thinking.
2. The mechanisms behind the design of organelle shape, mutual organelle movement, and precise mutual placement between the organelles are completely unknown. It has not been proven that proteins have a superior meaning for these mechanisms. We think that an information-carrying field recruited by the cells is responsible for building the organelles and other cellular organizations.
3. When the axon proliferates through growth *in vitro*, a symmetrical mirror image can be observed, indicating that cell movement and axon excrescence could be influenced by superior inside powers of organization able to steer the cytoskeleton organization of the cell. This could also be the case for the dendrites. We think that a superior control of the cytoskeleton through positional information causes cell movement and axon and dendrite excrescence of cells. To explain this protein activity, we chose to describe the cytoplasm as a liquid crystal phase.
4. In general, different cells and cell structures are made by the same molecules. The different ways of organization may therefore be due to varying information. We think the information necessary for this organization can be outlined as a pattern imprinted to the biological system. Therefore, we assume that a former undiscovered natural phenomenon exists behind the transmission of information for the cell structure. We call our explanation the holistic paradigm.
5. Information-transmitting interactions give the information for the organization to the cells so these can organize themselves through complex behavior. Through new information, these cells can be organized at a higher level. We think the information-directed self-organization in cells is able to explain cell ontogenesis.

## ACKNOWLEDGMENTS

These studies were supported by grants from IMK Almene Fond. Our research in quality of life has been approved by the Copenhagen Scientific Ethical Committee under number (KF)V.100.2123/91.

## References

1. Alberts, B., Johnson, A., Levis J., Raff, M., Roberts, K., and Walker, P. (1983) *Molecular Biology of the Cell.* Garland Science, Taylor & Francis Group.
2. Thorburn, W.M. (1915) Occam's razor. *Mind*, **24**, 287–288.
3. Thorburn, W. M. (1918) "The Myth of Occam's razor", *Mind*, 27, 345–353.
4. Schrödinger, E. (1958) *What is Life?* Cambridge University Press.
5. Buhse, H.E., Jr. and Williams, N.E. (1982) A comparison of cortical proteins in Tetrahymena vorax microstomes and macrostomes. *J. Protozool.* **29(2)**, 222–226.
6. Lee, J.J., Hutner, S.H., and Bovee, E.C. (1985) *Illustrated Guide to the Proto-zoa.* Allen Press, USA.
7. Purves, D. and Lichtman, J.W. (1985) *Principles of Neural Development.* Sinauer, Sunderland, MA.
8. Ventegodt, S., Hermansen, T.D., Flensborg-Madsen, T., Clausen, B., Nielsen, M.L., and Merrick, J. (2006) A theory for life, biological information, healing, and adult human metamorphosis for use in clinical holistic medicine VI: supracellular morphogenesis — the origin of biological and cellular order. Submitted to *TheScientificWorldJOURNAL.*
9. Cormack, D.H. (1987) *Ham's Histology*. J.B. Lippincott, Philidelphia.
10. Hammersen, F. (1985) *Histology. Color Atlas of Microscopic Anatomy*. Urban & Schwarzenberg, Baltimore-Munich.
11. Ventegodt, S., Andersen, N.J., Kromann, M., and Merrick, J. (2003) Quality of life philosophy II: what is a human being? *TheScientificWorldJOURNAL* **3**, 1176–1185.
12. Ventegodt, S., Andersen, N.J., and Merrick, J. (2003) Quality of life philosophy III. Towards a new biology: understanding the biological connection between quality of life, disease, and healing. *TheScientificWorldJOURNAL* **3**, 1186–1198.
